# Reply to Wostyn, P. Targeting the Cerebrospinal Fluid Compartment in Glaucoma: Still the Dark Side of the Moon? Comment on “Passaro et al. Glaucoma as a Tauopathy—Is It the Missing Piece in the Glaucoma Puzzle? *J. Clin. Med*. 2023, *12*, 6900”

**DOI:** 10.3390/jcm13051332

**Published:** 2024-02-27

**Authors:** Maria Laura Passaro, Francesco Matarazzo, Gianmarco Abbadessa, Antonio Pezone, Antonio Porcellini, Fausto Tranfa, Michele Rinaldi, Ciro Costagliola

**Affiliations:** 1 Department of Neurosciences, Reproductive Sciences and Dentistry, University of Naples “Federico II”, 80131 Naples, Italy; marialaura.passaro@unina.it (M.L.P.); fausto.tranfa@unina.it (F.T.); ciro.costagliola@unina.it (C.C.); 2 Pineta Grande Hospital, Castel Volturno, 81030 Caserta, Italy; francesco.matarazzo@unina.it; 3 Division of Neurology, Department of Advanced Medical and Surgical Sciences, University of Campania Luigi Vanvitelli, 80138 Naples, Italy; gianmarcoabbadessa@gmail.com; 4 Department of Biology, University of Naples “Federico II”, 80126 Naples, Italy; antonio.pezone@unina.it (A.P.); antonio.porcellini@unina.it (A.P.)

We are grateful to the author of the comment [1] who has kindly taken the time to comment on our review article [2]. The insightful commentary provided offers profound illumination for our paper, provoking thoughtful reflections on its content.

The in-depth exploration of the role of cerebrospinal fluid (CSF) in the progression of normal tension glaucoma is a contemporary and highly significant issue that deserves special attention.

Evidence in the literature increasingly recognizes the pivotal role of translaminar pressure difference (TPD) in the pathophysiology of normal tension glaucoma (NTG). Axon death in patients with NTG may be caused, at least in part, by an imbalance in the difference between intraocular pressure (IOP) and the pressure in the subarachnoid space (SAS) of the optic nerve. In 2015, a meta-analysis demonstrated that patients with glaucoma, including both NTG and high-tension glaucoma, exhibit elevated TPD compared to healthy individuals. Furthermore, in glaucoma patients, it was found that heightened TPD correlates with more pronounced structural alterations in the optic disc [3].

Recently, researchers have explored disrupted CSF dynamics within the context of optic nerve sheath compartment syndrome as a potential mechanism that may contribute to the development of glaucomatous optic nerve damage [4,5]. A difference in the concentration gradients between contrast-loaded CSF within the intracranial spaces and the optic nerve subarachnoid space in patients with NTG compared with healthy subjects has been demonstrated [4]. Moreover, individuals with NTG appear to exhibit a narrower optic canal on CT reconstructions when compared to healthy subjects [6]. CSF velocity flow ratios in the brain parenchyma, intracranial subarachnoid space, and the subarachnoid space surrounding the optic nerves were also evaluated using diffusion magnetic resonance imaging, showing a reduction in CSF flow ratios within the optic nerve SAS in NTG patients compared to control groups [7]. Finally, emerging evidence suggests that the pathophysiology of NTG involves not only CSF pressure, but also its content, as CSF is involved in the clearance of solutes and wastes from the optic nerve [8].

This evolving understanding emphasizes the intricate role that CSF dynamics may play in the development and progression of NTG, opening avenues for new therapeutic approaches targeting CSF flow modulation. Several questions about the role of CSF dynamics in NGT pathophysiology still await resolution. These lingering queries represent promising areas for further exploration and warrant additional research efforts to increase our understanding of these critical themes.
